# Calpain 2 Controls Turnover of LFA-1 Adhesions on Migrating T Lymphocytes

**DOI:** 10.1371/journal.pone.0015090

**Published:** 2010-11-30

**Authors:** Lena Svensson, Alison McDowall, Katherine M. Giles, Paula Stanley, Stefan Feske, Nancy Hogg

**Affiliations:** 1 Leukocyte Adhesion Laboratory, Cancer Research UK London Research Institute, London, United Kingdom; 2 Department of Pathology, Langone Medical Center, New York University, New York, New York, United States of America; New York University, United States of America

## Abstract

The immune cells named T lymphocytes circulate around the body fulfilling their role in immunosurveillance by monitoring the tissues for injury or infection. To migrate from the blood into the tissues, they make use of the integrin LFA-1 which is exclusively expressed by immune cells. These highly motile cells attach and migrate on substrates expressing the LFA-1 ligand ICAM-1. The molecular events signaling LFA-1 activation and adhesion are now reasonably well identified, but the process of detaching LFA-1 adhesions is less understood. The cysteine protease calpain is involved in turnover of integrin-mediated adhesions in less motile cell types. In this study we have explored the involvement of calpain in turnover of LFA-1-mediated adhesions of T lymphocytes. Using live cell imaging and immunohistochemistry, we demonstrate that turnover of adhesions depends on the Ca^2+^-dependent enzyme, calpain 2. Inhibition of calpain activity by means of siRNA silencing or pharmacological inhibition results in inefficient disassembly of LFA-1 adhesions causing T lymphocyte elongation and shedding of LFA-1 clusters behind the migrating T lymphocytes. We show that calpain 2 is distributed throughout the T lymphocyte, but is most active at the trailing edge as detected by expression of its fluorescent substrate CMAC,*t*-BOC-Leu-Met. Extracellular Ca^2+^ entry is essential for the activity of calpain 2 that is constantly maintained as the T lymphocytes migrate. Use of T cells from a patient with mutation in ORAI1 revealed that the major calcium-release-activated-calcium channel is not the ion channel delivering the Ca^2+^. We propose a model whereby Ca^2+^ influx, potentially through stretch activated channels, is sufficient to activate calpain 2 at the trailing edge of a migrating T cell and this activity is essential for the turnover of LFA-1 adhesions.

## Introduction

T lymphocytes circulate continuously in the blood with the purpose of monitoring the tissues for injury or infection. They use the integrin lymphocyte function–associated antigen-1 (LFA-1; αLβ2; CD11a/CD18) to migrate from the circulation across vessel walls into lymph nodes and other tissues [1], [2]. On the migrating cell, clusters of high affinity LFA-1 are organised into a mid-cell region of attachment termed the “focal zone” that corresponds to the lamellar region in other cell types [3], while intermediate affinity LFA-1 is expressed at the leading edge [4]. These two conformations of LFA-1 co-operate to bring about efficient T lymphocyte migration.

In migrating fibroblasts and endothelial cells, the cytosolic cysteine protease calpain is responsible for the turnover of integrin-mediated adhesions, promoting adhesion and spreading at the leading edge and deadhesion at the rear of the cell [5], [6]. Active calpain is considered to function by cleaving proteins that are constituents of adhesions [5], [6]. In particular, a calpain-insensitive mutant of talin prevented turnover of adhesions in fibroblasts [7]. There are two major calpain heterodimers, calpain 1(µ/I) and calpain 2(m/II) that share a subunit, calpain 4. Both forms have been described to function in migration [5], [6].

Calpain activity may be modulated, for example, by phosphoinositide/lipid binding [8], [9] or phosphorylation [10]. Phosphorylation of calpain 2 following epidermal growth factor receptor signalling occurs through the MAP kinase pathway involving ERK and MEKK1 and requires no overt increase in cellular Ca^2+^ [11], [12]. However the calpains are considered to be Ca^2+^ requiring enzymes and, in cell-free assays, calpain activation is Ca^2+^-dependent, requiring 2-75 µM for calpain 1 and 200–1000 µM Ca^2+^ for calpain 2 [5], [6]. How calpain activity is controlled in intact cells where the steady state levels of Ca^2+^ range between 50–300 nM requires further resolution [13].

For lymphocytes, the process of store operated calcium entry (SOCE) brings about Ca^2+^ influx following engagement of immunoreceptors such as the T cell or chemokine receptors [14], [15], [16]. Signalling through PLCγ leads to inositol-1,4,5-triphosphate (IP3) binding to the IP3R-expressing Ca^2+^ stores and subsequent Ca^2+^ release. The decrease of Ca^2+^ in the stores is sensed by stromal interaction molecule-1 (STIM1) which then activates the main calcium-release-activated-calcium (CRAC) channel composed of ORAI1 subunits resulting in Ca^2+^ influx from the extracellular space. The transient receptor potential (TRP) channel superfamily of >20 members comprises a second type of ion channel. The TRP channels have enormous diversity in terms of function and mechanisms of action, but their activity differs from that of the CRAC channel, for instance, in lacking Ca^2+^ selectivity; whether some TRP channels are activated by depletion of Ca^2+^ stores is still a matter of controversy [17], [18], [19], [20]. Finally, the influx of Ca^2+^ is modulated by potassium efflux through the outwardly rectifying K_v_1.3 and K_Ca_ channels that generates negative membrane potential and aids Ca^2+^ influx through the CRAC channel.

Previously we demonstrated a role for calpain in the adhesion of T lymphocytes to LFA-1 ligand intercellular adhesion molecule-1 (ICAM-1) [21]. It is known that calpain is involved in the migration of slow moving cells such as fibroblasts, but whether calpain makes a contribution to the migration of immune cells has been uncertain with reports of a suppressive role in myeloid cells [22] and a recent study in T lymphocytes indicating a null effect [23]. However other studies in T lymphocytes show calpain to make a positive contribution to adhesion and migration [24], [25], [26], [27]. It is possible that conflicting results are due to off-target effects of calpain inhibitors. In this study we have used the approach of siRNA knockdown, in addition to the most frequently used calpain inhibitor, calpeptin. We show that turnover of LFA-1 adhesions is controlled by calpain 2. Additionally we demonstrate that the activation of this calpain in T lymphocytes predominates at the trailing edge and is maintained by constitutive Ca^2+^ signalling.

## Results

### A role for calpain in LFA-1-mediated migration of T lymphocytes

**Figure 1:**
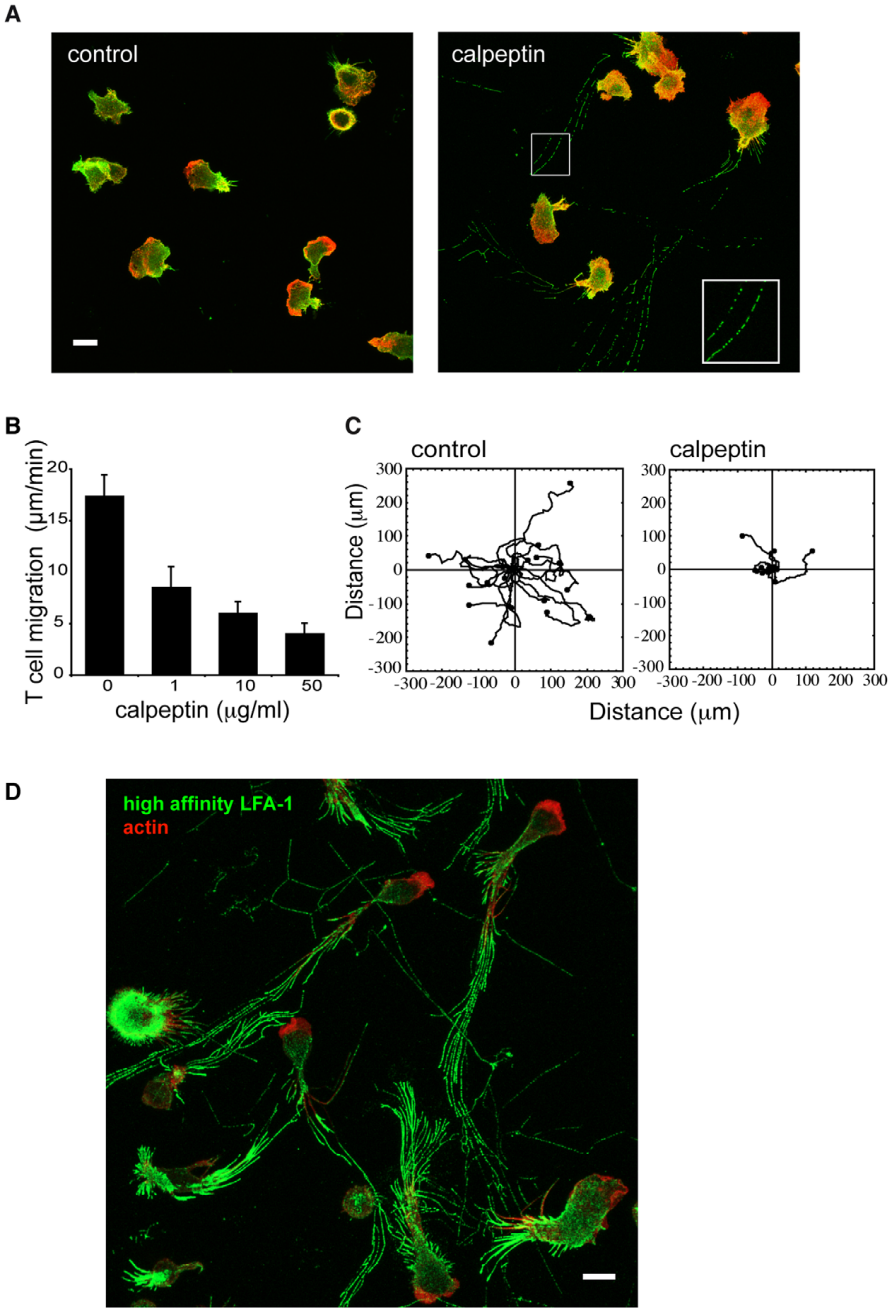
Calpain regulation of T cell migration. (**A**) T lymphoblasts were allowed to migrate for 15 min on ICAM-1 in the presence or absence of calpain inhibitor, calpeptin, before fixation. Total LFA-1 was detected using anti-LFA-1 mAb 38 and F-actin with fluorescently-labelled phalloidin. Panels are composite images of Z sections through the cells starting from the interface with ICAM-1. LFA-1 mAb (green), F-actin (red). Inset: higher magnification view of the LFA-1 trails shows distinct clusters of integrin. Scale bar = 10 µm. Images are representative of n = 4 experiments; (**B**) The calpain inhibitor, calpeptin, reduces the speed of migration on the LFA-1 ligand ICAM-1. For untreated and calpeptin-treated T cells, the speed was calculated for 20 cells over a 15 min period, n = 4; (**C**) Calpain regulation of T cell migration. Individual T cells display random migration when observed for 15 min with reduced migration following calpeptin treatment (50 µg/ml) (n = 20 T cells/experiment, n = 3 experiments); (**D**) Detail of LFA-1 clusters detected with mAb 24 in trails of calpain-inhibited migrating T cells after 45 min. The panel is a composite image of Z sections through the T cells. LFA-1 mAb 24 (green), F-actin (red), Scale bar = 10 µm. Images are representative of n = 4 experiments.

To address whether calpain is involved in the migration of T lymphocytes, the cell permeable calpain inhibitor, calpeptin, that targets the active site of calpain [28] was added to T lymphoblasts (T cells) migrating on ICAM-1. In untreated T cells, an F-actin rich leading edge (red) was observed with LFA-1 expression (green) displaying graded distribution towards the rear of the cell (**Fig. 1A**). In the presence of calpeptin, this expression pattern remained constant. However LFA-1 was also observed in punctate adhesions behind each cell (see inset) (**Fig. 1A**).

Calpeptin was titrated to test whether blocking calpain activity affected T cell migration. At a concentration of 50 µg/ml, calpeptin reduced T cell speed by 76.6±5.2% (control-17.4±2.0 µm/min; 50 µg/ml calpeptin- 4.1±1.0 µm/min) (**Fig. 1B**). Another general inhibitor of calpain, PD150606, that targets the Ca^2+^ binding site [28], had a similar effect (control- 14.4±0.8 µm/min; 50 µg/ml PD150606- 3.0±0.3 µm/min). The random pattern of migration of individual T cells was also prevented by calpeptin (**Fig. 1C**).

Calpeptin-treated T cells that were fixed after 45 min of migration and stained with high affinity LFA-1 mAb 24 displayed trails of the integrin, highlighting the individual cell trajectories (**Fig. 1D**). Although the cells undergo loss of LFA-1, it is apparent that contractile forces are still able to move them forward at least for a limited time period. Together the data show that active calpain has an essential role in the migration of T cells at the level of LFA-1 adhesion turnover and that the adhesions include high affinity LFA-1.

### Calpain is required for turnover of LFA-1 adhesions at the trailing edge of the T cell

**Figure 2:**
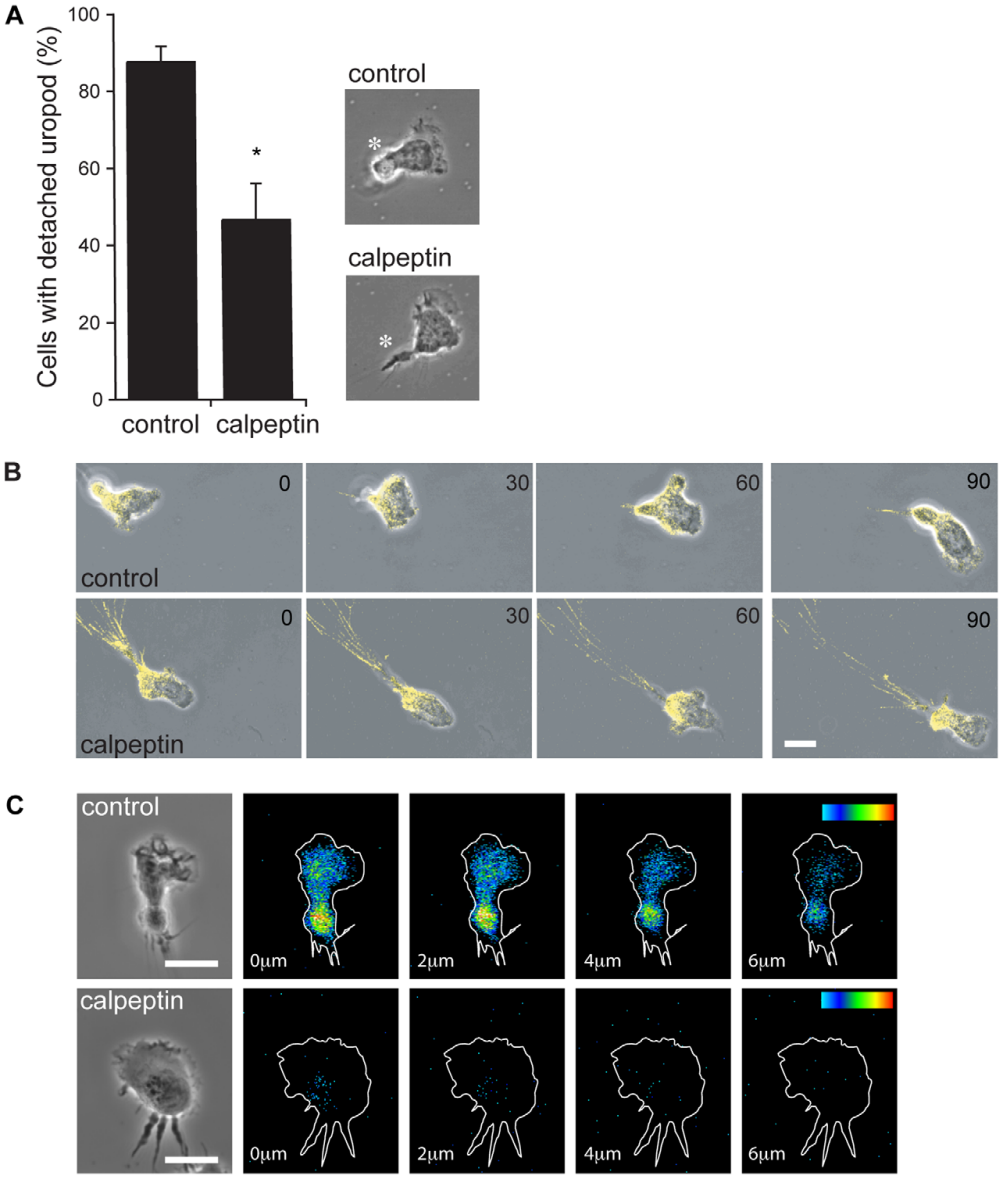
The effect of calpain inhibition and location of active calpain in T cells. (**A**) Effect of calpain inhibition on T cell morphology. The proportion of untreated and calpeptin-treated T cells with a detached uropod (n = 50 per data set). Representative images of untreated and calpeptin-treated T cells are shown with the uropod and trailing edge respectively indicated by a white asterisk. *P<0.05; (**B**) Effect of calpain block on distribution of LFA-1 and morphology of migrating T cells over time (seconds). T cells were labelled with Alexa488 conjugated-Fab' fragments of a non-function affecting anti-LFA-1 mAb YTH81.5 (green), then allowed to migrate on ICAM-1-coated substrate ± calpeptin. Images are representative of n = 4 experiments; (**C**) Distribution of active calpain in T cells loaded with calpain substrate CMAC,*t*-BOC-Leu-Met and adhered to ICAM-1 ± calpeptin. Z section images were taken through migrating cells at 2 µm intervals from the level of T cell/ICAM-1 interface. Pseudo-color glow scale (red, highest to blue, lowest activity). Scale bar = 10 µm, images are representative of n = 4 experiments.

We next investigated how calpain inhibition altered the morphology of migrating T cells. Control T cells displayed the classic “hand-mirror” shape with a spreading lamella at the leading edge and a detached, upwardly projecting uropod at the trailing edge (**Fig. 2A**). Following calpeptin treatment, T cells continued to spread, but lost the distinctive uropod structure and became elongated with the trailing edge attached to the substratum.

To study the dynamics of this calpain-inhibited effect in more detail, we performed live cell imaging with T cells that were labelled with Alexa488-conjugated Fab' fragments of a non-function blocking LFA-1 mAb. The distribution of LFA-1 on the migrating T cell was again skewed towards the rear in both calpeptin-treated and untreated cells. Control T cells migrated efficiently, detaching their trailing edges and maintaining uropods (**Fig. 2B**; **Movie S1**). In contrast, as calpeptin-treated cells migrated on ICAM-1, thin LFA-1-expressing extensions developed from the back of the cell. Eventually fresh adhesions and forward movement caused sufficient membrane tension to rupture the link with LFA-1 and release the trailing edge of the cell (**Fig. 2B** **; Movie S2**).

By using the calpain substrate CMAC,t-BOC-Leu-Met (CMAC) we could directly visualize calpain activity in migrating T cells. The cleaved fluorescent product was highly concentrated at the rear of the migrating T cell and at the interface with ICAM-1(**Fig. 2C**). Treatment of T cells with calpeptin resulted in the loss of CMAC fluorescence and therefore calpain activity. Calpain inhibition again caused the appearance of thin extensions at the rear of the cell. Thus active calpain is concentrated at the rear of the cell and at the interface with ICAM-1.

Together these findings support the idea that calpain is involved in the turnover of LFA-1 adhesions at the rear of the cell. When calpain activity is blocked, the cell has difficulty in detaching so that, as the front of the cell moves forward, there is trailing edge elongation and loss of the uropod structure.

### Calpain is activated by extracellular Ca^2+^

In cell-free systems activation of calpain is Ca^2+^-dependent, but a role for Ca^2+^, or indeed another divalent cation, in stimulating calpain in intact immune cells such as T cells has not been examined. Stimulating levels of cation could originate from several sources: extracellular influx via cell membrane ion channels, release from the intracellular Ca^2+^ stores located in the endoplasmic reticulum (ER) or from both sources [14], [16], [29].

**Figure 3:**
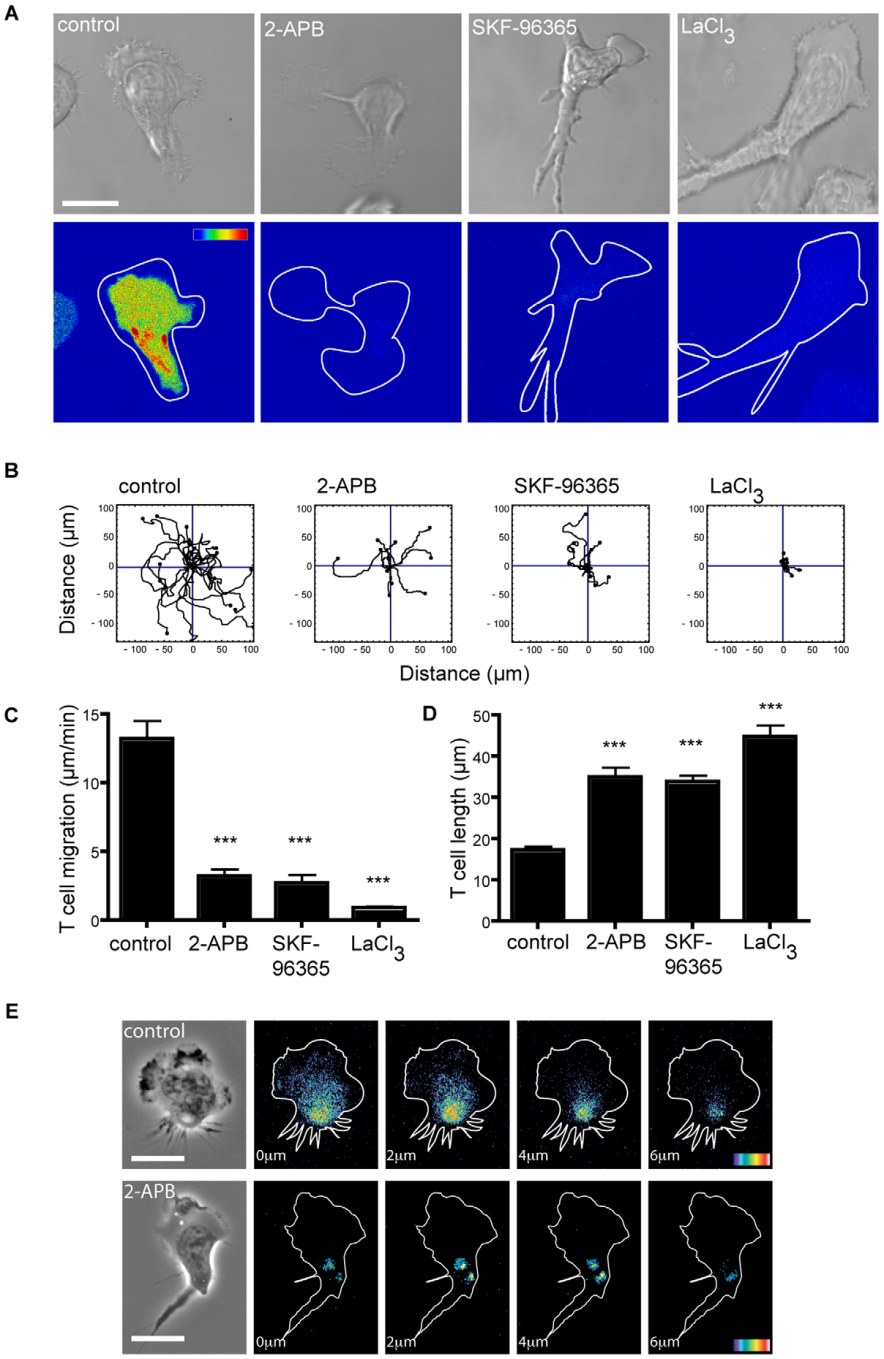
Effect of Ca^2+^ channel inhibitors on calpain activity and T cell migration. Effect of ion channel inhibitors 2-APB (50 µM), SKF-96365 (100 µM), LaCl_3_ (2 mM) on the following (**A–D**): (**A**) Calpain activity as detected by expression of calpain substrate CMAC,*t*-BOC-Leu-Met. Pseudo-color glow scale (red, highest to blue, lowest activity). Outline of DIC image shown as a white tracing. Scale bar = 10 µm. Images are representative of n = 4 experiments; (**B**) Individual T cells show random migration following observation for 20 min (n = 15 cells, n = 3 experiments); (**C**) Overall speed of T cell migration (n = 15 cells). *** *P*<0.001; (**D**) Cell length of migrating cells, n = 25 cells. *** *P*<0.001; (**E**) Ca^2+^ levels observed in T cells polarized on ICAM-1 before and after treatment with 2-APB. Panels show Z sections from the level of the ICAM-1/T cell interface up through the cell at 2 µm intervals. Pseudo-colour glow scale (red, highest to blue, lowest activity). Scale bar = 10 µm; images are representative of n = 3 experiments.

We examined the effect of blocking the membrane ion channels using pharmacological agents to see how the lack of ion influx might affect calpain activity. We tested SKF-96365, an imidazole used to block receptor-mediated Ca^2+^ entry [30], LaCl_3_, a cation channel inhibitor used to block CRAC and TRP channels [14], [31] and 2-APB, an general inhibitor of intracellular Ca^2+^ signaling [32]. When calpain activity was visualized by microscopy, the three inhibitors blocked formation of fluorescent CMAC and caused an elongated morphology (**Fig. 3A**). These features were associated with a reduction in migration, either when quantified by the tracking of individual cells (**Fig. 3B**) or collectively as overall speed (Control, 13.2±1.3 µm/min; SKF-96365, 2.70±0.64 µm/min (80.0±3.5% decrease); LaCl_3,_ 0.9±0.13 µm/min (93.5±0.4% decrease); 2-APB, 3.2±0.55 µm/min (76.5±2.5±2% decrease) (**Fig. 3C**).

When the effect of the inhibitors on the migratory phenotype was viewed microscopically, they all caused cell lengthening (**Fig. 3D**). The attached cells extended at their leading edges until the increase in tension caused a rupture of the LFA-1 adhesions (data not shown). The reduction in speed and elongated cell morphology caused by the inhibitors was explained by the restraining action of the more firmly attached trailing edge on the active front of the cell that continued to move forward.

The use of the inhibitors suggested that the activity of a membrane ion channel was essential for maintenance of both active calpain and migration of T cells. As calpain activity *in vitro* has been demonstrated to be Ca^2+^-dependent, it was important to test whether a relevant ion channel was delivering Ca^2+^ into the cell. To locate cellular Ca^2+^, T cells migrating on ICAM-1 were loaded with the Ca^2+^ binding Fluo-4 dye and examined by fluorescent time-lapse confocal microscopy. The cells displayed elevated Ca^2+^ that was confined predominantly to the rear of the cell and at the interface with ICAM-1 (**Fig. 3E**). A link between the membrane ion channel and maintenance of the Ca^2+^ level at the rear of the cell was demonstrated by treating T cells with 2-APB resulting in a substantial decrease in visible Ca^2+^ (**Fig. 3E**).

### The ORAI1-expressing CRAC channel is not involved in calpain activation and T cell migration

**Figure 4:**
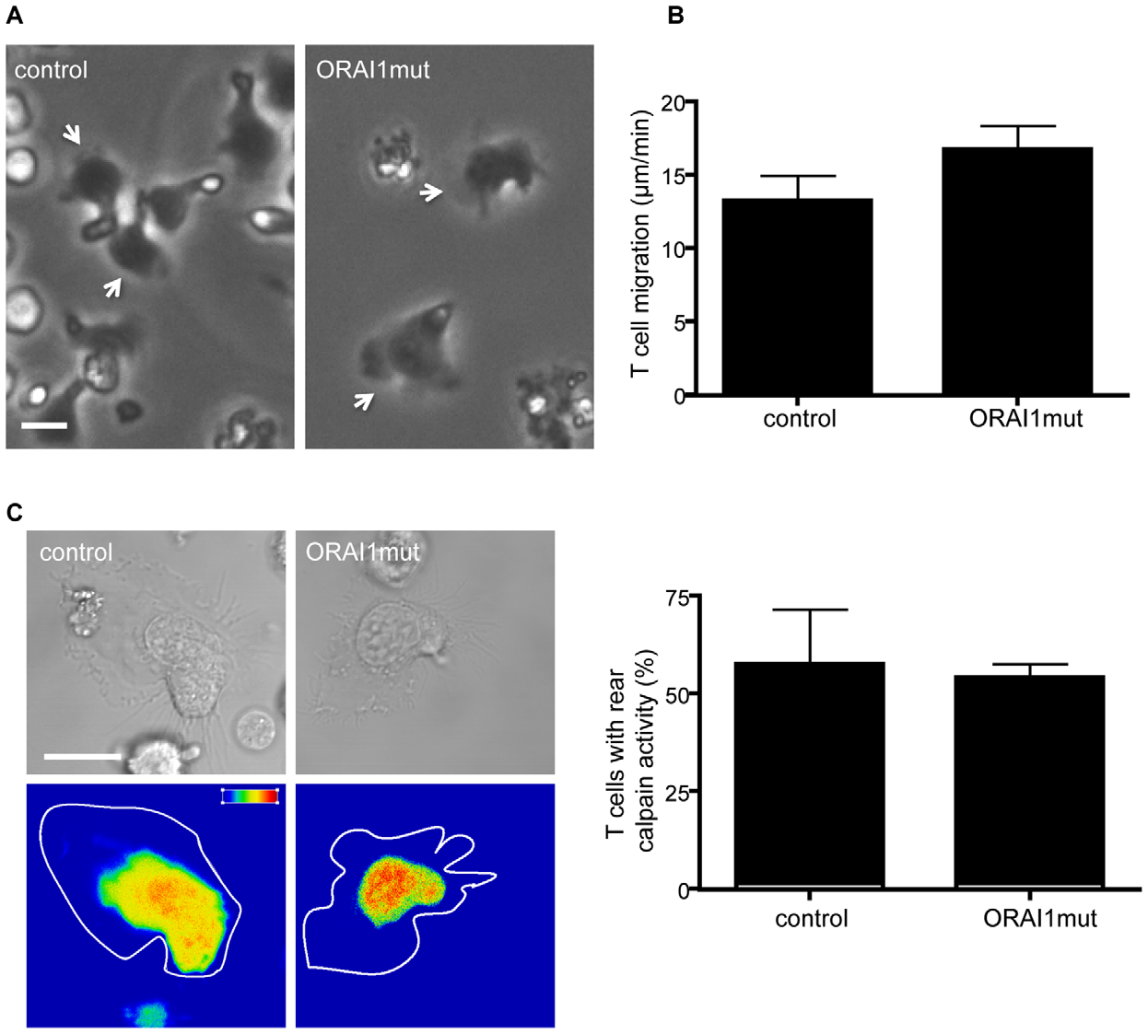
Investigation of ORAI1 mutant T cell calpain activity and migration. (**A**) Morphology of control and ORAI1 mutant T cell lines migrating on ICAM-1. White arrow indicates the leading edge of the T cell; n = 3 complete sets of experiments for each cell line; (**B**) Overall speed of migration (n = 12 T cells); (**C**) Calpain activity of individual control and ORAI1 mutant T cell lines as detected by expression of calpain substrate CMAC,*t*-BOC-Leu-Met. Pseudo-color glow scale (red, highest to blue, lowest activity). Outline of DIC image shown as a white tracing. Scale bar = 10 µm; (**D**) Proportion of total T cells expressing CMAC activity (n = 15 cells).

The CRAC channel in human T cells contains the ORAI1 protein as a pore forming subunit and is essential for SOCE in T cells and other immune cells [14], [16], [33]. To ask whether this Ca^2+^ conducting channel was responsible for calpain activation and the migration of T cells, we used a human T cell line derived from a immunodeficient patient homozygous for a non-functional mutated ORAI1-R91W protein and compared its migratory potential with a similarly maintained wild type control T cell line [34]. Both control and mutant T cells migrated with similar morphology (**Fig. 4A**) and speed (**Fig. 4B**) indicating that lack of a functioning ORAI1-expressing CRAC channel did not adversely affect T cell migration. Importantly when calpain activity was investigated, active enzyme was present in a similar proportion of both control and ORAI1 mutant T cells and at a similar level in the rear of the cells (**Fig. 4C, D**). These findings indicated that the major CRAC channel plays neither a role in migration nor in the general level of calpain activation observed in T cells.

### Calpain 2 is active at the trailing edge of the migrating T cell

**Figure 5:**
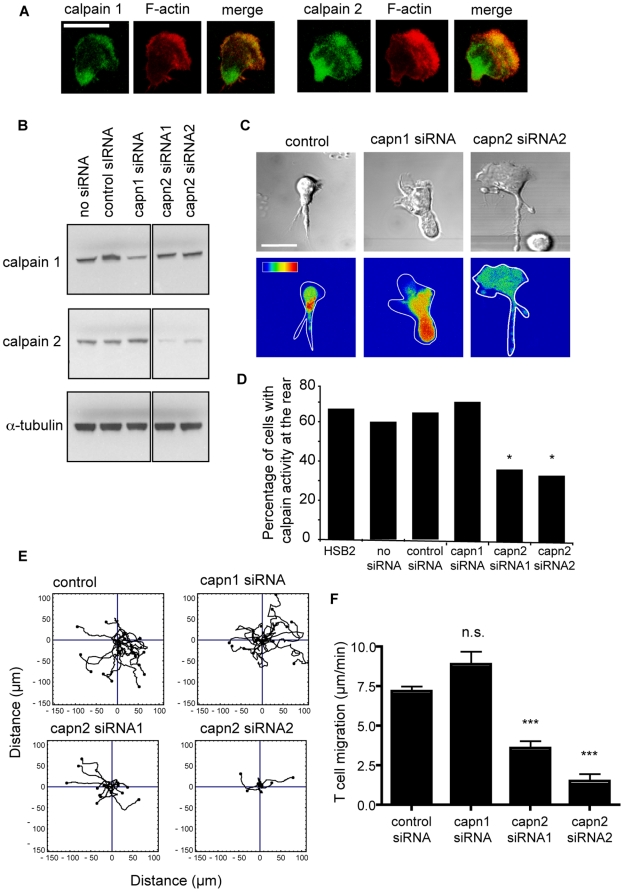
Calpain activity at the rear of the T cell is due to calpain 2. (**A**) Immunofluorescent staining of fixed T cells migrating on ICAM-1. The images are composites of Z sections taken through the cell. Calpain mAbs = green; F-actin = red. Scale bar = 10 µm; images are representative of n = 3 experiments; (**B**) Western blotting of the HSB2 T cell lysates following calpain 1 and 2 siRNA knockdown, α-tubulin represents loading control; n = 4 experiments; (**C**) Calpain activity in the HSB2 T cells following calpain 1 and 2 siRNA knockdown as detected by expression of calpain substrate CMAC,*t*-BOC-Leu-Met; pseudo-color glow scale (red, highest to blue, lowest activity). Scale bar = 10 µm; (**D**) Quantification of HSB2 cells displaying calpain activity following siRNA knockdown of calpain 1 or 2, n = 2–5 experiments, *P<0.05; (**E**) Individual T cells displaying random migration observed for 20 min and (**F**) quantification of overall speed of migration (n = 15 cells, representative of n = 3 experiments), *** *P*<0.001.

Since there are two major isoforms of calpain, 1 and 2, expressed in migrating T cells, the next question was which isoform might be active at the trailing edge. As might be expected for cytosolic enzymes, both calpain 1 and calpain 2 were distributed throughout the cell, from the leading edge, where they overlapped with F-actin, to the trailing edge (**Fig. 5A**).

To identify which calpain isoform was active at the rear of the cell, the individual calpains were specifically knocked down in HSB2 cells (capn 1 siRNA by 75%; capn 2 siRNA1 by 86%, capn 2 siRNA2 by 85%) (**Fig. 5B**). It was noted that reduction in one calpain isoform did not affect the expression level of the other. Knockdown of calpain 2, but not calpain 1 nor control siRNA-treated cells, inhibited calpain activity at the trailing edge of the migrating T cell showing active calpain 2 is concentrated in this region (**Fig. 5C,D**). Finally to demonstrate a role for the calpain 2 isoform in T cell migration, we showed that knockdown of calpain 2, but not calpain 1, inhibited T cell migration at both the level of single cell tracking (**Fig. 5E**) and general cell speed (**Fig. 5F**).

In summary, although calpeptin is considered a specific inhibitor of the calpains, siRNA knockdown of calpain is more selective and allowed discrimination between the two calpains isoforms. We have therefore been able to show that, in spite of the general distribution of both calpain 1 and calpain 2 within the T cell, it is calpain 2 that is active at the rear of the T migrating cell.

## Discussion

In this study we show that calpain 2 has an essential role in releasing the LFA-1 adhesions of migrating T lymphocytes and that this calpain is maintained in an active state by Ca^2+^ ion channel activity. Inhibition of calpain 2 by calpain inhibitors and by siRNA silencing, causes reduced speed of migration by interfering with LFA-1 detachment at the trailing edge of the migrating T cell. The T cell can however shed integrin in the absence of efficient adhesion turnover, potentially through tension generated by trailing edge contraction. Over time, this results in trails of released LFA-1 adhesions that highlight the cell's trajectory.

There are conflicting findings over the role of calpains in integrin-mediated adhesion events in T cells. Our previous study and others indicate that calpain has a positive role in terms of promoting LFA-1- and β1- mediated integrin adhesion [21], [24], [25], [26], [27]. However in a recent report, no LFA-1 related adhesion defects were observed in T cells from mice where the calpain 4 subunit shared by calpain 1 and calpain 2 was conditionally deleted [23]. Previous studies, and also this present one, have made use of calpain inhibitors raising the possibility of non-specific effects. However we have also used siRNA knockdown to specifically reduce levels of both calpain 1 and 2 and observe specific effects of active calpain 2 on the migratory behavior of T cells. Other possibilities that might be considerd are that differences exist in calpain usage between mouse and human T cells, that the lack of calpain 4 was compensated for in generating active calpain 1 or 2 and finally there might be functional interplay between calpain 1 and 2 as has been reported previously [35] with still-to-be-explored functional consequences.

We show here that the clusters of T cell adhesions containing high affinity LFA-1 require a calpain-dependent step to deadhere. Consistent with these results are single particle tracking studies showing that calpain is responsible for releasing the immobile active form of LFA-1 on the membrane [26]. There is also a requirement for a calpain-mediated step when adhesion occurs under conditions of shear flow mimicking the circulation [36]. This is most relevant when ICAM-1 levels are low and turnover of LFA-1 is required to release old adhesions and form new ones.

Another issue is that T cells express both calpain 1 and calpain 2 and immunostaining indicates that both isoforms are distributed throughout the T cell. A question is why it is calpain 2, rather than calpain 1, that mediates the release of the LFA-1 adhesions. A major controlling factor may be the exact location of the calpains within the cell. Although the calpains have traditionally been considered to be cytosolic enzymes, there is evidence that calpain 2 is associated with cellular membranes through phosphoinositide binding, and this could give greater access to membrane bound ion channels [9], [37].

Of relevance is the level of Ca^2+^ required to activate calpain 2. Steady state intracellular Ca^2+^ levels range between 50–300 nM, whereas in cell free assays calpain 2 is activated by 200-1000 µM levels of Ca^2+^ [13]. We show here that intracellular Ca^2+^ is highest at the T cell membrane interface with ICAM-1. In terms of activating intracellular calpain-2, Ca^2+^ could be delivered locally at the membrane in a sufficiently high concentration. Ca^2+^ levels up to 100 µM have been recorded within 20 nm of Ca^2+^ channels and this may be sufficient to maintain calpain 2 activity [38], [39]. As a consequence, calpain-2 may well be colocalized with a Ca^2+^ channel to ensure the high, localized Ca^2+^ concentrations that are required for its activity.

Our findings demonstrate an essential requirement for a Ca^2+^-conducting ion channel in regulating both calpain 2 activity and LFA-1-mediated migration. The channel appears to be providing a constitutive or “tonic” signal as the T cells require no added stimulus in order to migrate. A key question is how this activity is initiated and maintained. Cell migration is known to be affected by stretch-activated calcium channels [40], [41]. The activity of such a channel, most highly expressed at the rear of the polarized cell, would provide an explanation for maintenance of the Ca^2+^ levels and calpain 2 activity.

The major ion channel that delivers Ca^2+^ into the cell is the ORAI1-CRAC channel that is activated by depletion of Ca^2+^ from the ER stores. It is of interest that it is not required for calpain activity or migration in T cells. Another candidate Ca^2+^ channel in T cells is TRPM7, a non-selective cation channel that is permeable to Ca^2+^ and also to Mg^2+^ and is an atypical TRP channel in expressing a C terminal α-kinase [42], [43], [44]. It is associated with calpain in fibroblast focal adhesions [31], [45] and there is some evidence that it can act as a mechanosensitive channel that is activated by shear stress of laminar flow [46]. Taqman assay revealed low levels of TRPM7 in T lymphoblasts and HSB2 cells (data not shown). However, the role of TRPM7 for migration and calpain activity in human T cells could not be tested in the present study because, despite much effort, it proved impossible to reduce TRPM7 levels using the siRNA approach (data not shown). TRPM7 remains a good candidate as it is associated with Ca^2+^ influx and migration in other cell types [47], [48].

The distinction in terms of function between the ORAI1-CRAC channel and the calpain 2-activating ion channel described in this study may have to do with compartmentalisation of Ca^2+^ influx. Our evidence suggests that calpain 2 is activated close to the T cell membrane and this is consistent with the finding that LFA-1-dependent Ca^2+^ entry can occur without the release of intracellular Ca^2+^ [49]. On the other hand, ORAI1-CRAC channels are activated upon Ca^2+^ store depletion giving rise to a more global elevation of Ca^2+^ within the cell and this distribution may have other consequences. For example Ca^2+^ influx through the CRAC channel plays a major role in controlling transcription of immune mediators such as cytokines in response to immunoreceptor engagement and resulting in activation of the calcineurin/NFAT pathway [14], [16].

In summary, we show in this study that calpain-2 regulates the release of LFA-1 adhesions in migrating T lymphocytes, and is maintained in a constitutively active state dependent on influx of Ca^2+^. The lack of involvement of the major ORAI1-expressing CRAC channel has been unexpected and points to a clear distinction in the roles of Ca^2+^ delivering ion channels in T lymphocyte function.

## Materials and Methods

### Inhibitors, antibodies and other reagents

LFA-1 mAbs 38 and YTH 81.5 were produced at Cancer Research UK and Fab' fragments of mAb YTH81.5 directly conjugated to Alexa-488 fluorochrome as previously described [3]. Calpain 2 mAb 3A11D12 was kindly provided by Dr. Mitsuchi Inomata [50]. The following mAbs were purchased: calpain 1, Calbiochem; calpain 2, Research Diagnostics; α-tubulin, Sigma. The calpain inhibitors were purchased from Merck Biosciences: calpeptin (50 µg/ml unless stated) and PD150606 (50 µg/ml). The following reagents were purchased as indicated: 2-APB, Merck Bioscience; lanthanum chloride (LaCl_3_), Sigma; SKF-96365, Sigma; pre-designed siRNAs for calpain 1 (ID 146578), calpain 2 (ID 112796 and 145947), and siRNA control #2 were all from Ambion; CAPN1 13 and 10, CAPN2 1 and 6 and negative control were from Qiagen.

### Cell isolation, culture and electroporation

Peripheral blood mononuclear cells were prepared from single donor leukocyte buffy coats (National Blood Service) and T cells expanded as previously described and used between days 10 and 14 [21]. The human CD3^-^ leukemic T cell line, HSB2 was maintained in RPMI 1640/10% FCS [51]. Human polyclonal, non-transformed T cell lines from an ORAI1-mutated patient and wild type control were generated as previously described [34] and maintained in RPMI/10% FCS in the presence of IL-2.

For siRNA knockdown experiments, HSB2 T cells line were washed twice in OptiMEM+GlutaMAX (Invitrogen) and electroporation was performed on 2×10^7^ cells with 200–400 nM siRNAs using a Gene Pulser with Capacitance Extender (Bio-Rad) set at 960 µFD and 260 mV as previously described [3]. The siRNA-electroporated cells were maintained in RPMI 1640/10% FCS for 24 h prior to use. The efficiency of knockdown was evaluated by Western blot analysis for calpain with quantification using NIH image 1.63 software.

### Microscopy

#### i. Video microscopy

35 mm glass bottomed petri dishes (MatTek Corp., Ashland Mass. USA) or or µ-slides VI (Ibidi, Thistle Scientific Ltd, Glasgow, UK) were coated overnight at 4°C with 3 µg/ml ICAM-Fc then blocked for non-specific binding with 2.5% BSA/PBS for 1 h at RT as previously [3]. T cells were washed with HBSS and 10^5^ cells added to dishes containing HBSS/20 mM HEPES. Cells migrated for 15 min at 37°C prior to initiation of the experiment. Images were captured with an Olympus MTV3 Inverted microscope and a 20× lens or a Zeiss Axiovert 135TV Inverted microscope and a 63× lens plus AQM^2001^ Kinetic Acquisition Manager software (Kinetic Imaging Ltd., Bromborough, UK). Cells were tracked using Motion Analysis software (Kinetic Imaging Ltd.) and data analysed using a Mathematica notebook (Wolfram Research Europe Ltd, Long Hanborough, UK) developed by Daniel Zicha (Cancer Research UK, London).

#### ii. Live cell imaging of LFA-1 distribution

T cells (2×10^5^/ml) were labelled with Alexa488-conjugated YTH81.5 Fab' fragments (10 µg/ml) for 15 min at 37°C, washed and added to ICAM-1Fc-coated MatTek dishes as described above. T cells were allowed to migrate for 20 min ±50 µg/ml calpeptin, then images were taken at 10 second intervals using a Zeiss Axiovert microscope and a LSM-510 laser scanning system.

#### iii. Live cell imaging of calpain activity

T cells or HSB2 T cells at 5×10^6^ cells/ml were labelled with the calpain substrate CMAC,*t*-BOC-Leu-Met (20 µM; Invitrogen) for 10 min at 37°C [52], washed and added to ICAM-1Fc-coated MatTek or µ-slide VI dishes as described above. Images were taken using a Zeiss Axiovert microscope with Blue Diode and a LSM-510 laser scanning system within 20 min.

#### iv. Confocal microscopy

T lymphoblasts ± calpeptin, exposed to ICAM-1Fc coated coverslips for 40 min at 37°C, were fixed with 3% formaldehyde in HBSS for 20 min at RT. Coverslips were incubated with 10 µg/ml of LFA-1 mAb in HBSS containing 0.25% BSA for 1 h at RT. Cells were permeabilised with 0.1% Triton-X-100, for 4 min at RT then incubated with 2.5 units/ml Alexa 546-phalloidin and Alexa 488-goat anti-mouse IgG (1∶200; Invitrogen) for 1 h at RT. Finally, coverslips were mounted in Mowiol antifadent (Merck Biosciences) and images collected on a Zeiss Axioplan microscope with a LSM510 laser scanning system.

#### v. Live cell imaging of T cell Ca^2+^

T cells were labelled with Fluo-4 (1 mM; Invitrogen) plus 10% w/v Pluronic 127 (Invitrogen) for 60 min at 37°C, washed and added to ICAM-1Fc-coated dishes (MatTek) as described above. Cells were visualized by phase contrast and the Ca^2+^ flux by excitation at 480 nm and emission at 516 nm. Images were taken at 30 sec intervals using a Zeiss Axioplan microscope with a LSM510 laser scanning system.

### SDS-PAGE and Western blotting of calpain siRNA treated HSB2 cell extracts

HSB2 T cells were lysed at 5×10^7^/ml for 20 min on ice in 50 mM Tris pH7.4 containing 150 mM NaCl, 1% Triton X-100, 20 µg/ml PMSF and a complete protease inhibitor cocktail tablet (Roche). The lysate was microfuged for 15 min to remove insoluble material. Proteins were separated under reducing conditions by SDS-PAGE. After transfer to nitrocellulose membrane and incubation with antibodies, bound antibody was detected with HRP-conjugated sheep anti-mouse Ig (GE Healthcare, UK) and ECL Western blotting detection reagents (GE Healthcare).

### Statistical Analysis

The significance of the migration assays was determined using 2 Way Anova (Mathematica notebook (Wolfram Research) further developed by Daniel Zicha (Cancer Research UK)). The significance of other analyses (Fig. 2A, 3D) was tested using the unpaired Student t test (GraphPad Prism software version 5 for Macintosh computers). Fisher's Exact Test was used to the analyse the pooled data from 2-5 experiments represented in Fig. 5D. The following significant differences are as indicated: *, *P*<0.05; **, *P*<0.01; ***, *P*<0.001.

## Supporting Information

Movie S1 The distribution of LFA-1 on human T lymphoblasts migrating on ICAM-1. LFA-1 is labelled with Alexa488-conjugated Fab' of non-function-blocking mAb YTH81.5.
(AVI)

Movie S2 The distribution of LFA-1 on human T lymphoblasts migrating on ICAM-1 following treatment with calpeptin at 50 µg/ml. LFA-1 is labelled with Alexa 488-conjugated Fab' of non-function–blocking mAb YTH81.5.
(AVI)
